# A lung cancer research agenda that reflects the diverse perspectives of community stakeholders: process and outcomes of the SEED method

**DOI:** 10.1186/s40900-018-0134-y

**Published:** 2019-01-11

**Authors:** Carlin L. Rafie, Emily B. Zimmerman, Dawn E. Moser, Sarah Cook, Fatemeh Zarghami

**Affiliations:** 1 Department of Human Nutrition, Foods and Exercise, Virginia Polytechnic Institute and State University, 321 Wallace Hall (0430), 295 West Campus Drive, Blacksburg, Virginia 24061 USA; 2 Center on Society and Health, Virginia Commonwealth University, 830 East Main St., Suite 5032, P.O. Box 980212, Richmond, Virginia 23298-0159 USA; 3 Martinsville, Virginia 24112 USA; 4 Vanderbilt Institute for Clinical and Translational Research (VICTR), Vanderbilt University Medical Center, 2525 West End Ave, Nashville, TN 37203 USA

**Keywords:** Stakeholder engagement, Research question development, Community based participatory research

## Abstract

**Plain English summary:**

There is a need for methods that engage lay people and other stakeholders, such as patients and healthcare providers, in developing research questions about health issues important to them and their communities. Involving stakeholders helps ensure that funding goes to research that addresses their concerns. The SEED Method engages stakeholders in a systematic process to explore health issues and develop research questions. Diverse groups of stakeholders participate at three levels: as collaborators that lead the process throughout, as participants who use their expertise to develop the questions, and as consultants who provide additional perspectives about the health topic. We used the SEED Method to engage 61 stakeholders from different socioeconomic and professional backgrounds to create research questions on lung cancer outcomes. Participants included cancer patients and caregivers, healthcare providers and administrators, and policymakers from a rural Virginia community. They developed causal models that diagrammed factors that influence lung cancer outcomes and the relationships between them. They used these models to develop priority research questions. The questions reflect the participants' diverse perspectives and address different areas of inquiry related to lung cancer outcomes, including access to care, support systems, social determinants of health, and quality of care. Participants felt well prepared to perform the project tasks because they had the opportunity to review lung cancer information, receive causal model and research question development training, and participate in facilitated group activities. The SEED Method can be used in a variety of settings and applied to any health topic of interest to stakeholders.

**Abstract:**

**Background**

Engagement of stakeholders in prioritization of health research can help ensure that funding is directed to research that reflects their concerns and needs. The Stakeholder Engagement in quEstion Development and Prioritization (SEED) Method is a multi-stakeholder methodology that uses principles of community engagement and causal modeling to develop health research questions that reflect the priorities of patients, clinicians, and other community stakeholders. We conducted a demonstration of the SEED Method to generate research questions on lung cancer outcomes, and to evaluate the process, outcomes, and effectiveness of the method for generating a research agenda that reflects diverse stakeholder perspectives.

**Methods**

The SEED Method engages community members at three levels: collaboration, participation, and consultation. We conducted a demonstration project from November, 2015 to July, 2016, in a rural Virginia community that was experiencing a significant disparity in lung cancer outcomes. A community research team led the project and selected three distinct stakeholder groups (Topic groups, TG) for participatory engagement in analysis of the health issue, causal modeling, and research question development. We evaluated the quality of stakeholder engagement and compared TG causal models and research questions to evaluate the diversity of stakeholder perspectives resulting from the methodology.

**Results**

The resulting research agenda poses questions on how a broad range of topics including access to care, support systems and coping mechanisms, social determinants of health, and quality of care impacts lung cancer outcomes. Participants felt well prepared for the tasks they were asked to perform due to the technical trainings and facilitated modeling and question development activities that are part of the SEED Method. The causal models and research questions developed by the Topic Groups reflected the diverse perspectives of the stakeholders.

**Conclusions**

The SEED Method has the potential to generate relevant stakeholder-centered research agendas on a variety of health-related topics, and to create community capacity for sustained research engagement.

**Electronic supplementary material:**

The online version of this article (10.1186/s40900-018-0134-y) contains supplementary material, which is available to authorized users.

## Background

End users of health research are increasingly being engaged throughout the research process. Involvement of those impacted by health issues in the identification and prioritization of research topics allows inclusion of their unique experiential understanding and ensures that research priorities reflect their concerns. [1–3] It also provides opportunities for research that is more valid, relevant, accepted, and sustainable. [3] Various methods for engaging patients and clinicians in topic generation have been used. A systematic review of 148 studies revealed a variety of engagement techniques, including Delphi exercises and face-to-face meetings, and found that all methods engaged participants directly and repeatedly. [4] The majority of studies had clinicians and patients working separately, and most used formal methods for reaching decisions including voting, scoring, individual rating, and consensus conferences. [4]. Best practice recommendations for topic generation processes include ensuring collaboration between patients and clinicians, peer consultations, data analysis, and consensus-building. [5] Recent examples exist of methods that apply these practices, using iterative processes for research topic generation and prioritization with multi-stakeholder research advisory groups and priority setting partnerships [6, 7]. The SEED (Stakeholder Engagement in quEstion Development and Prioritization) Method was developed to fill a gap in stakeholder engagement strategies that are community driven and use participatory methods to engage clinician and patient stakeholders in health research question development and prioritization. [8, 9] We conducted a demonstration of the SEED Method with community stakeholders in a rural community in southern Virginia to develop a research agenda relevant to lung cancer outcomes.

A cancer needs assessment conducted by the authors (CR and DM) in this community in 2014 identified a disparity in lung cancer mortality as a significant health issue. [10] Lung cancer is the third most commonly diagnosed cancer and the leading cause of cancer death among both men and women of all races and ethnicities in the United States. [11] The national age-adjusted lung cancer death rate in 2017 was 44.7, compared to 45.5 in Virginia, and 73.1 in the target community for the SEED demonstration. [12] The 5-year survival rate remains very low at only 18.1%. [13] Racial and socioeconomic lung cancer disparities exist and involve complex, interconnected influences of the living environment, behaviors, sociocultural factors, and biology of individuals. [14] The SEED Method is designed to consider the multiplicity of influences on health outcomes using a socio-ecological approach and was conducted as a follow-up to the findings of disparate lung cancer outcomes in the community.

We implemented SEED from November 2015 to July 2016 to explore factors influencing lung cancer outcomes and develop a stakeholder-driven research agenda. We report on the process and outcomes of the SEED Method applied in this context and the effectiveness of the method at generating a research agenda that reflects diverse stakeholder perspectives.

## Methods

The SEED Method was piloted through funding from the Patient Centered Outcomes Research Institute (PCORI) in a previous project. [15] The method was elaborated by Zimmerman based on a participatory conceptual modeling process that was piloted in two previous projects with colleagues at the Virginia Commonwealth University Center on Society and Health. [16, 17] SEED is founded on community-based participatory research (CBPR) principles and uses causal modeling to facilitate research question development by community stakeholders. It engages stakeholders at three levels; (1) collaborative engagement of a community research team to lead the project throughout the research, (2) participatory engagement with stakeholders working within distinct groups to generate and prioritize research questions, and (3) consultative engagement with stakeholders who add additional perspectives and experiential knowledge to inform the process. [15]

**Fig. 1:**
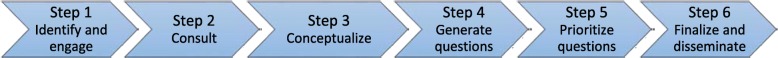
Steps in the SEED Method (adapted from Zimmerman et al. Am J Prev Med 2017;53(1):123–129)

We implemented the SEED Method in a six-step process that first engaged collaborative stakeholders as a community research team. This team managed the project across a period of nine months. The community research team identified targeted groups of stakeholders, called Topic groups (TG), for participatory engagement in the analysis of the health issue and research question development. Other stakeholders, called SCAN participants, were engaged in a consultative fashion to gather additional perspectives about lung cancer outcomes in the local context and inform the TGs as they conceptualized the issue through creation of causal models. Each TG used their model to generate and prioritize research questions. The final step was a review of the scientific literature related to the prioritized research questions by university- and community-based researchers and graduate students to focus the research agendsa on identified research gaps. (Fig. 1) We evaluated the quality of stakeholder engagement in SEED and compared causal models and research questions between TGs to assess the effectiveness of stakeholder engagement in generating distinct research priorities. [15, 18] The study was approved by the Virginia Tech IRB, and all participants provided informed consent.

### Implementation of the SEED Method in Southern Virginia

#### The research team (collaborative engagement)

A community research team, Engaging Martinsville (EM), led the project from launch through dissemination. The project coordinator of the previous cancer needs assessment (a community resident) joined the research team and facilitated recruitment of ten additional community members through notifications to community organizations, multimedia advertising, and individual communication. Personal experience with and/or interest in lung cancer and the ability to commit the time to the research team activities were the primary criteria for selection. Individual communication was the most effective means of recruitment. Research team members were paid an hourly wage for the duration of the project. The EM team was diverse in experience, age, and race (36% white, 55% black) and was predominately female (73%) with an educational attainment above high school (91%). Three of the team members left before the end of the project due to relocation and changes in work schedule.

EM was involved in all aspects of project management and met weekly throughout the project. Principles of CBPR guided implementation of SEED. [19, 20] In particular, a collaborative and equitable partnership guided the engagement of stakeholders, empowering each member to express their opinions through processes of shared accountability and decision making. These same principles characterized the engagement of TG members in their work.

EM determined the composition of the TGs through a process designed to ensure diversity in experience and perspectives. Briefly, EM used decision aids (SEED stakeholder identification matrices) to identify priority stakeholder subgroups within three general categories: (1) patients and caregivers, (2) healthcare providers, and (3) others. After brainstorming a list of subgroups in each category, the team identified selection criteria that they used to rank the subgroups in order of importance. EM used a voting system to determine the final composition of the TGs. Three TGs were selected. EM then identified appropriate recruitment locations and used fliers, direct communication, and newspaper advertisements to recruit participants. Interested participants were screened for eligibility during a phone conversation. Individuals were advised during the screening of their eligibility status. Newspaper advertisements and direct communication were the two most successful recruiting methods.

#### The topic groups (participatory engagement)

The three TGs selected were: (1) lung cancer patients and caregivers (LCP/C, *n* = 7), (2) non-physician clinical care providers (CCP, *n* = 8) involved in lung cancer patient care, and (3) access influencers (AI, *n* = 6) able to influence access to detection, treatment, and survivorship care. Selection criteria included subgroups with a high prevalence of lung cancer and those residing within the community for the LCP/C, providers with greater than five years of healthcare experience for the CCP, and significant community involvement and an interest in lung cancer for the AI. TG participants received a stipend for their participation. Three TG members dropped out of the project (one from each group) due to time constraints. Similar to the EM team, TG members were predominantly female (76%) with greater than a high school education (91%). TGs had greater representation from adults older than 65 years (28%) and people of Caucasian race (62%) than the EM team.

### Topic group activities

#### Orientation to the health issue

TGs each met on seven occasions and worked separately throughout the process. During the first four, 90-min meetings, TGs were oriented to their task and provided information about lung cancer by the EM team. Each TG then identified additional stakeholders (SCAN participants) from whom they wanted information to inform their task. SCAN participants were interviewed or participated in focus groups, and received a stipend for their participation. Ten key informant interviews (physicians, health care service providers, health and lung cancer advocacy organizations, and patients), and four focus groups (lung cancer patients, caregivers, faith leaders, and non-clinician providers) were conducted by the EM Team. Two university-based project members conducted content analysis of the transcripts and summarized the recurring themes. The EM team reviewed the summaries and discussed them with the TGs.

#### Causal modeling and question development

Creation of the causal models and research questions occurred during the last three, 180-min TG meetings. Causal models describe the causal mechanisms of a system, and are widely used to propose the interrelationships between dependent and independent variables and moderating and mediating factors. These models can be useful for guiding formulation of research questions and directing future research. [21, 22] TGs received training on causal model development and then participated in a facilitated process of brainstorming factors affecting lung cancer outcomes, positioning those factors in relation to lung cancer outcomes, and depicting causal pathways between factors. Final causal models were created through group discussion and consensus on the factors and their positions within the model. Each TG compared their own model with that of the other two groups.

After a brief training on research question development, TGs drew on the models and the information acquired throughout the project to create research questions around lung cancer outcomes. Question prompts were employed to help generate diverse questions, focusing participants on causes, impacts, verification of relationships, and new directions for thinking about lung cancer outcomes. Each member developed research questions, which were discussed by the group. The TGs then prioritized their research questions through a discussion and multi-voting process. [23] The four highest priority research questions were chosen by each TG to form the final, 12-question research agenda.

#### Identifying knowledge gaps

A review team of university and community researchers and graduate students conducted a literature review of the 12 research questions to identify existing evidence and target research gaps. The original research questions were reworded and the review findings were added as additional sub-questions. EM presented the final research agenda to the TGs and the community.

#### Evaluation

We evaluated the SEED Method process using questionnaires, activity and observation logs, after action reviews, and participant interviews. Questionnaires were created for this project, but drew on questionnaires published by others. [24–27] Questionnaires included a personal information questionnaire, group readiness and group dynamics questionnaires completed by the EM team and TGs, and satisfaction questionnaires. Activity and observation logs, as well as the after action reviews, were used to evaluate and improve the SEED process. Activity logs were completed by the activity facilitators after completion of the three stakeholder selection matrices by the EM team, and the causal modeling, question development and prioritization activities of the Topic Groups. Observation logs were completed by a member of the SEED administrative team during these same activities. After action reviews were conducted with TGs after key activities. End of project interviews conducted with EM members evaluated personal and community impact of the project.

To evaluate the diversity of perspectives contributed by each TG, we compared the number and content of factors across the causal models. Two EM members independently grouped factors into categories and reached consensus on category names and factor classification through discussion. Presence and placement of categories and the complexity of linkages between them were also compared.

Research questions were independently grouped into query domains by two EM members to facilitate comparison. Consensus on the domain names, and research question grouping was reached through team discussion. We evaluated unique and duplicate question query domains between the TGs and the relationship of research questions to corresponding factors in the respective causal models for each TG.

## Results

### Group readiness and dynamics

Group dynamics is one of four dimensions of CBPR research that can influence project outcomes. [28] We collected group readiness surveys at the beginning of the study and group dynamics at study conclusion. There was consensus among EM and TG participants that they were ready to share openly during the project, their group represented the community, and the project would have personal and community benefit. A majority of respondents felt the group was networked to the community and understood its needs. EM and TG members strongly agreed that there were positive group dynamics with open communication and respect regardless of demographics or socioeconomic status. The majority experienced personal growth and gained new skills through the project. Although most were satisfied with facilitation and decision-making processes, some were ambivalent in this area (Table 1).

**Table 1:** Engaging Martinsville and Topic Group Member Responses: Group Readiness and Group Dynamics

Group Readiness	Respondents^a^	Strongly Agree	Agree	Disagree/ Strongly Disagree
1. I am open to learning new skills throughout this project	Research team	72.7%	27.3%	0.0%
	Topic groups	81.3%	18.8%	0.0%
2. I am willing to share my opinions and life experiences with other group members.	Research team	81.8%	13.2	0.0%
	Topic groups	87.5%	12.5%	0.0%
3. I have a clear picture of the time it will take to be involved in this project	Research team	63.6%	36.4%	0.0%
	Topic groups	75.0%	19.0%	0.0%
4. I understand my role within this project.	Research team	54.5%	45.5%	0.0%
	Topic groups	68.8%	31.3%	0.0%
5. I am willing to mentor and be mentored throughout this project.	Research team	81.8%	18.2%	0.0%
	Topic groups	75.0%	18.8%	6.3%
6. I believe this project will benefit patients and stakeholders.	Research team	72.7%	27.3%	0.0%
	Topic groups	81.3%	18.8%	0.0%
7. I believe this project will benefit my community.	Research team	90.9%	9.1%	0.0%
	Topic groups	87.5%	12.5%	0.0%
8. I think I will benefit from participating in this project.	Research team	100.0%	0.0%	0.0%
	Topic groups	81.3%	18.8%	0.0%
Group Diversity				
9. Our group reflects the diversity of our community.	Research team	54.5%	45.5%	0.0%
	Topic groups	68.8%	31.3%	0.0%
10. Our group members are networked to the community and understand its history, politics, and needs.	Research team	27.3%	72.7%	0.0%
	Topic groups	50.0%	37.3%	12.6%
Group Dynamics				
1. I can talk openly and honestly at team meetings.	Research team	81.8%	18.2%	0.0%
	Topic groups	82.4%	17.6%	0.0%
2. Team members respect each other’s point of view even if they might disagree.	Research team	100.0%	0.0%	0.0%
	Topic groups	82.4%	17.6%	0.0%
3. My opinion is listened to and considered by other team members.	Research team	100.0%	0.0%	0.0%
	Topic groups	82.4%	17.6%	0.0%
4. All team members are made to feel welcome regardless of income, age, race, gender, or education level.	Research team	100.0%	0.0%	0.0%
	Topic groups	88.2%	11.8%	0.0%
5. It takes too much time for the team to reach decisions.^b^	Research team	9.1%	18.2%	82.8%
	Topic groups	N/A	N/A	N/A
6. Everyone in the team has a voice in the decisions.^b^	Research team	90.9%	9.1%	0.0%
	Topic groups	N/A	N/A	N/A
7. Some members of the team hold onto their ideas too tightly. ^b^	Research team	18.2%	0%	81.8%
	Topic groups	N/A	N/A	N/A
Benefits/Costs of Participation				
8. Participating in this project has provided personal growth for me.	Research team	90.9%	9.1%	0.0%
	Topic groups	90.9%	9.1%	0.0%
9. Since starting to work on this project, my skills and knowledge have increased.	Research team	81.8%	18.2%	0.0%
	Topic groups	81.8%	18.2%	0.0%
Understanding of the SEED Method				
10. I understand my role in the SEED project	Research team	81.8%	18.2%	0.0%
	Topic groups	76.5%	23.5%	0.0%
Group decision making process		Very satisfied/ satisfied	Unsure	Dissatisfied/very dissatisfied
11. Satisfaction with meeting facilitation	Research team	81.8%	18.2%	0.0%
	Topic groups	76.5%	23.5%	0.0%
12. Satisfaction with how team works	Research team	90.9%	9.1%	0.0%
	Topic groups	82.4%	17.6%	0.0%
13. Satisfaction with decision making process^b^	Research team	81.8%	18.2%	0.0%
	Topic groups	N/A	N/A	N/A
14. Satisfaction with way team deals with problems	Research team	81.8%	18.2%	0.0%
	Topic groups	70.6%	29.4%	0.0%

^a^Research Team (*n* = 11), and Topic Groups (*n* = 16 for questions 1–10, *n* = 18 for group dynamics questions 1–14)

^b^These questions were irrelevant to the Topic groups, and were not included on the abbreviated group dynamics questionnaire

Responses to open-ended questions about experiences with the project fell into four categories: respectful sharing of ideas, forming new friends and networks, knowledge gain, and helping the community. TG members expressed satisfaction with the process overall, and EM team members appreciated the group diversity, closeness, and the satisfaction working for their community. There were no major conflicts among members, but the meeting schedule, length and slow pace of some meetings were areas for improvement.

### Causal models

Causal model comparison showed that the clinical care provider (CCP) TG had the greatest number of factors (*n* = 51) and factor categories (*n* = 19), followed by the lung cancer patient and caregiver (LCP/C) group (*n* = 37 & *n* = 14), and the access influencer (AI) group (*n* = 36 & *n* = 13). A total of 90 factors were identified, of which sixty-four were unique to a single TG, and twenty-six were found in two or more of the models (Table 2).

**Table 2:** Causal Model Factor Comparison

Unique Factors			Factors Common to Two or More Groups
Lung Cancer Patients and Caregivers (LCP/C)	Clinical Care Providers (CCP)	Access Influencers (AI)	
Body weight	Access to care	Addictions	Affordability of care
Community support	Alternative health care	Availability of medications	Age
Exercise	Cancer stage	Delayed or misdiagnosis	Availability of care
Faith in God	Community involvement	Drinking alcohol	Communication ability
Fitness	Co-morbid conditions	Family size	Coordination of care
Follow up care	Computer literacy	Fear	Denial
Food quality	Coping skills	Immigration status	Getting information
Having a regular doctor	Culture	Inherited conditions	Hope
Household cleaning	Education on treatment options	Insurance status	Income
Leisure activities	Family dysfunction	Lack of education on cancer symptoms	Literacy
Maintaining independence	Financial support	Mindfulness	Mental health
Pain management	Genetic testing	Religion	Pain
Place	Health literacy	Social programs	Physical health
Quitting smoking	Housing	Specialized care	Positivity
Second-hand smoke	IQ		Prayer
Sense of control	Marital status		Quality of care
Trust in doctor	Pollution		Religious practices
Willingness to take risks	Procrastination		Smoking/quitting
	Quality of death		Social policies
	Quality of life values		Social values
	Race/ethnicity		Sources of information
	Resistance to medical model		Support for caregivers
	Resource management		Support from family
	Risk factors		Transportation
	Screening		Use of care
	Self-care		Occupational health/work conditions
	Stress		
	Transportation		
	Trust		
	Urban/rural		
	Will to live		
	Willingness to ask for help		

**Fig. 2:**
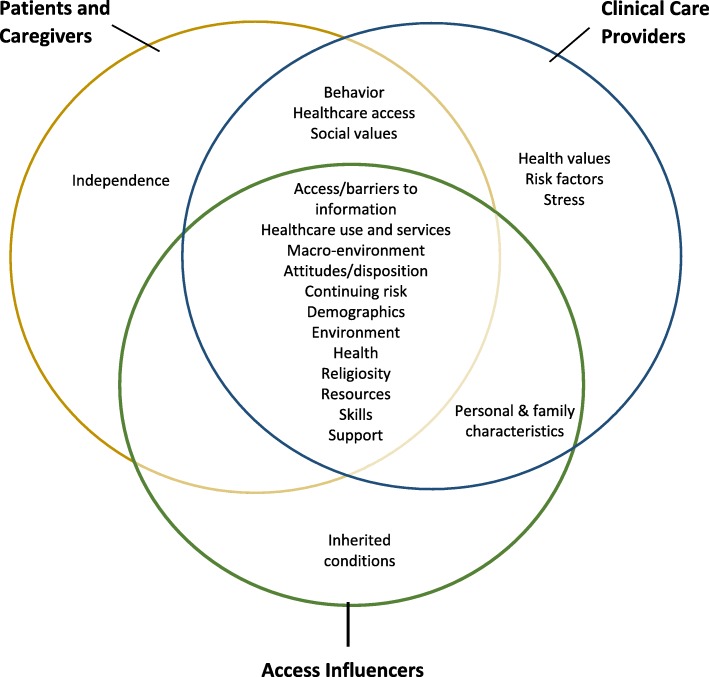
Causal Model Factor Categories by Topic Group

**Fig. 3:**
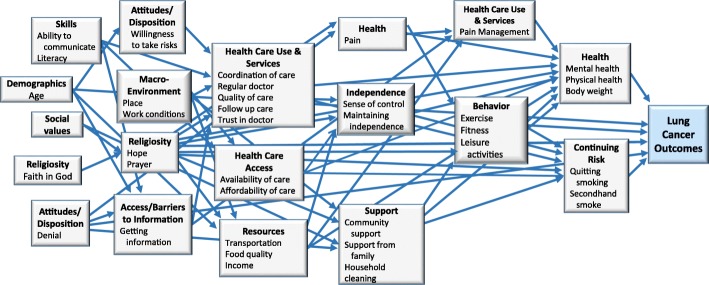
Lung Cancer Patient and Caregiver Topic Group Causal Model

There were twenty-one categories of factors. Of these, five were unique to one causal model and sixteen were common in two or more causal models. The CCP group had three unique categories (health values, risk factors, and stress), and there was one unique category in the LCP/C (independence) and AI groups (inherited conditions) (Fig. 2). The CCP model had the greatest number of connections between factor (68 connections), followed by the LCP/C model (54 connections), and the AI model (31 connections). Overall, the CCP model showed the greatest complexity, followed by the LCP/C model, and lastly the AI model. The LCP/C model is illustrated in Fig. 3.

### Research questions

Each TG created between twenty-one and twenty-seven questions. Questions were prioritized and each group selected the top four for inclusion in the final research agenda, for a total of twelve questions. Grouping the final questions into query domains resulted in four domains: Barriers/Access to Care, Support Systems/Coping Mechanisms, Social Determinants of Health, and Quality of Care. The domain, Quality of Care, was unique to the CCP TG, and Support Systems/Coping Mechanisms was unique to the LCP/C TG. All four of the research questions generated by the AI TG fell into the Barriers/Access to Care query domain. The research agenda with the original questions is found in Table 3. The final research agenda with the refined questions and sub-questions generated after the literature review is included in Additional file 1.

**Table 3:** Final Topic Group Research Questions

Cancer Patient and Caregiver Topic Group	
1. What are the factors of patients' faith (for example, knowing what happens when you die, feeling of peace or seeing family members again) and knowing family and community are praying for them; and how does this affect lung cancer outcomes? Does it reduce stress and does it change their outlook? (Support Systems/Coping Mechanisms) 2. Does living in a poverty stricken area versus an area of higher standards affect lung cancer? (Social determinants of health) 3. Why is there no help available through the healthcare system to discuss alternative treatments, natural choices, diet and nutrition? (Barriers/Access to care) 4. If society required insurance companies to offer free annual checkups with x-rays would lung cancer be diagnosed earlier? (Barriers/Access to care)	
Clinical Care Providers Topic Group	
5. If we could affect perceptions of care at local hospitals and providers, would it change outcomes? (Quality of care) 6. Would more assistance navigating the healthcare system improve outcomes? (Quality of care) 7. If we could improve the general health of the population would it affect lung cancer outcomes? (Social determinants of care) 8. If patients knew more about hospice goals and palliative care, would it affect treatment decisions and outcomes? (Barriers/Access to care)	
Access Influencers Topic Group	
9. If screenings and early detection occur, does MHMHC, Memorial Hospital of Martinsville and Henry County (local hospitals), have the resources and technology to provide efficient and expedited methods to diagnose and stage? (Barriers/Access to care) 10. If healthcare insurance coverage was standardized for diagnosis and treatment of cancer, would lung cancer outcomes become better? (Barriers/Access to care) 11. Would paid FMLA (Family Medical Leave Act) legislation for caregivers benefit lung cancer outcomes and how? (Barriers/Access to care) 12. If the Primary Care Physician recommended regular screening, the patient met qualifying guidelines, and the cost was covered, would they have the screening? What are the reasons why not? Would it change the lung cancer outcomes? (Barriers/Access to care)	

## Discussion

The SEED method is unique in that the health issue of interest is community-identified and the method uses causal modeling to inform research question development. It involves diverse stakeholders, including clinicians, patients, and others, in order to generate a diverse research agenda. Similar to other engagement models that separate clinicians and lay people, stakeholder groups work independently to produce and prioritize research questions. The process is conducted within a single community primarily with stakeholders without prior research experience. It engages stakeholders repeatedly through a series of in-person meetings. This contrasts with other methods that solicit input, often electronically, from a large number of different stakeholders in a multi-step process of research topic generation, reduction, and final prioritization, as with priority setting partnerships. [7] Notably, unlike other methods whose primary focus is on clinical care research questions, the SEED Method generates research questions on a wide range of issues related to the health topic.

Sixty-one participants of varying socioeconomic, educational, and professional backgrounds contributed to the lung cancer outcomes research agenda. The process whereby the EM team selected the TGs ensured diverse viewpoints on the problem. This is illustrated by the fact that individual representatives within each group fell into five of the seven stakeholder categories outlined in the 7Ps Framework for Stakeholder Engagement, including patients and the public, providers, purchasers, payers, and policy makers. [29] Each TG worked independently to highlight their unique experiences and to ensure that questions from each group were included in the final research agenda. Topic groups comprised of similar stakeholders helped facilitate group cohesion and open expression, and aimed to avoid power differentials that are common among mixed patient and provider groups.

We evaluated the quality of stakeholder engagement and compared the final products (causal models and research questions) developed by the different stakeholder groups to assess the effectiveness of the SEED Method in facilitating distinct stakeholder contributions. [4, 18] Our comparison of the TG causal models and research questions illustrates the unique perspectives that each brought to the health issue. A large proportion of factors (71%) in the causal models were unique to individual TGs, and the position of factor categories and number of connections between them varied between groups.

TG research questions fell into unique query domains and addressed distinct issues. For example, the LCP/C TG prioritized a research question about the intersection of faith and lung cancer outcomes (see Table 3). “Faith in God” was a unique factor in their causal model and they had unique discussions exploring the relationship of faith to coping with lung cancer, decision making and risk taking, and lung cancer outcomes. The ‘Religiosity’ category was positioned early in their model and contained ten connections to other factor categories, highlighting its importance from their perspective. This question fell into the query domain Support Systems/Coping Mechanisms, which was unique to this TG.

In a similar way, the Quality of Care query domain was unique to the CCP TG. The two research questions in this domain related to unique factors in the group’s causal model:

“Would more assistance navigating the healthcare system improve outcomes?” (Unique Factor: education on treatment options)

“If we could affect perceptions of care at local hospitals and providers, would it change lung cancer outcomes?” [Unique Factor: trust]

CCP had unique discussions on the need for specialized navigation services for patients with limited knowledge of cancer treatments, and the impact of negative perceptions and distrust of local healthcare on healthcare seeking behavior. Timeliness of lung cancer diagnosis and treatment were identified as important factors in lung cancer outcomes.

Finally, all four questions of the AI TG fell in the query domain, Barriers/Access to Care. The occupational focus of AI members on facilitating care access is reflected in their prioritization of research questions that address this domain.

Stakeholders define engagement as the active decision-making of committed stakeholders about a problem that is meaningful to them through a process of respectful interactions where everyone’s opinions are heard and carry weight. [30] The positive responses on the surveys are evidence that the SEED Method fostered equitable engagement and avoided many of the pitfalls of group dynamics.

A lack of technical training and capacity building in stakeholder engagement methodologies for collaborative research question development are common limitations. [31–33] Time to review data and discuss ideas, along with trainings in causal modeling and research question development that are part of SEED, address these limitations. The majority of stakeholders in this project felt well prepared for the tasks they were asked to perform.

## Limitations

The process length and time commitment were areas for improvement. We completed project activities (excluding the literature review) in nine months; TG activities occurred in months three through six. A shortened timeline could broaden the application of the method. A SEED Method toolkit has been developed that accommodates varying timelines according to project-specific objectives. Although our findings indicate a high degree of stakeholder satisfaction with the SEED process and effective stakeholder engagement, these results may vary depending on the context, experience, and relationships of university-community research teams.

It is important that a dissemination plan for the research agenda be part of stakeholder engagement projects and systems in order to link resulting research priorities to potential funders. This has been the process in multiple studies and public-clinician partnerships [5–7], and was part of our process. The project findings and final research agenda were disseminated to the community through two public presentations. We also presented the final research agenda to health researchers at two state universities, resulting in a doctoral research project conducted in the community evaluating patient and physician barriers to lung cancer screening. A number of the research questions are under review by AHRQ’s Effective Healthcare Program for an evidence review to help inform patient, clinician, and health system decision making. [34] In addition, the local hospital has taken several actions in response to the project, including implementation of a physician education campaign to increase lung cancer screening referral and systems to increase access to low dose CT to vulnerable populations.

## Conclusion

The SEED Method effectively engaged community stakeholders in the development of a patient-centered research agenda to address lung cancer outcomes. Participants with diverse viewpoints brought a range of perspectives on the social and environmental factors affecting health behaviors, decision making, and health outcomes. The SEED Method has the potential to generate stakeholder-centered research agendas on a variety of health-related topics and to create community capacity for sustained research engagement.

## Additional file

Additional file 1: Final Stakeholder Developed Research Agenda on Lung Cancer outcomes. The final lung cancer outcome research agenda with the refined questions and sub-questions generated after the literature review. (PDF 112 kb)

## Abbreviations

AI: Access influencers
CBPR: Community-based participatory research
CCP: Non-physician clinical care providers
EM: Engaging Martinsville
LCP/C: Lung cancer patients and caregivers
SCAN: Consultative stakeholders
SEED: Stakeholder Engagement in Research Questions Development and Prioritization
TG: Topic Group
