# Supplementary material for: A lung cancer research agenda that reflects the diverse perspectives of community stakeholders: process and outcomes of the SEED method
Source: Res Involv Engagem. 2019 Jan 11;5:3. doi: 10.1186/s40900-018-0134-y (PMC6330432; doi:10.1186/s40900-018-0134-y)
Supplement: Supplementary file 1 — Final Stakeholder Developed Research Agenda on Lung Cancer outcomes. The final lung cancer outcome research agenda with the refined questions and sub-questions generated after the literature review. (PDF 112 kb) [file 40900_2018_134_MOESM1_ESM.pdf]

Final Stakeholder Developed Research Agenda on Lung Cancer Outcomes

Barriers/Access to Care

1. **Why is there no help available through the healthcare system to discuss alternative treatments, natural choices, diet and nutrition?**
    - ❖ What are the barriers to communication about alternative medicine treatments with lung cancer patients within the healthcare system and how can they be addressed?
    - ❖ Which complementary and alternative medicine (CAM) modalities are effective at addressing the symptom and quality of life needs of lung cancer patients, and improving survival rates?
    - ❖ How can healthcare facilities be structured to facilitate the incorporation of CAM modalities into the treatment plans for lung cancer patients across the care continuum?
    - ❖ How can accurate information about effective CAM modalities be disseminated to lung cancer patients?
2. **If society required insurance companies to offer free annual checkups with x-rays would lung cancer be diagnosed earlier?**
    - ❖ Would free lung cancer screening for all people who meet current screening eligibility criteria in the US result in increased screening, earlier diagnosis and increased lung cancer survival? How would this vary by socioeconomic status?
    - ❖ Would free lung cancer screening for people over 74 years in the US result in earlier diagnosis, increased survival rates, and reduced mortality from lung cancer?
    - ❖ What employer based incentives could increase lung cancer screening among eligible employees.
3. **If patients knew more about hospice goals and palliative care, would it affect treatment decisions & outcomes?**
    - ❖ How would a more accurate knowledge of hospice goals and services affect lung cancer patient/caregiver treatment decisions & end of life outcomes?
    - ❖ How can prognostic information be communicated to cancer patients in an individualized, timely fashion that positively impacts end of life decisions? Which educational models are most effective at improving healthcare provider communication about prognosis and effectively help patients and families transition from curative to palliative/hospice care?
    - ❖ What cancer care models and initiatives effectively increase the timely communication of end of life options to patients with advanced cancer? Can palliative care programs be effective bridge programs to hospice? What is the impact on patient/caregiver hospice knowledge, treatment decisions, and length of hospice stay?
    - ❖ What are the barriers to timely entry into hospice for patients of lower socioeconomic status, racial/ethnic minorities, and residents in urban centers?

- ❖ What would be the impact of medical insurance policies that allow treatment during hospice enrollment on quality of life, length of hospice enrollment, and medical care cost?

4. **If screenings and early detection occur, does MHMHC, Memorial Hospital of Martinsville and Henry County (local hospitals) have the resources and technology to provide efficient and expedited methods to diagnose and stage?**

- ❖ How can hospitals, in healthcare limited communities, accurately anticipate and address the increased technology and human resource needs for efficient diagnosis, staging and treatment of lung cancer with increased screening?
- ❖ What are the best models of care to provide an interdisciplinary approach for lung cancer diagnosis, staging and treatment, while maintaining a financially viable program?
- ❖ What is the impact of location of residence on healthcare referral patterns, lung cancer treatment, and outcomes? How does this vary by race/ethnicity?
- ❖ What is the impact of regionalization of lung cancer care on lung cancer outcomes of patients in rural communities? What viable transportation models exist to support a regionalized health system?

5. **If healthcare insurance coverage was standardized for diagnosis and treatment of cancer, would lung cancer outcomes become better?**

- ❖ Would lung cancer outcomes improve across all socioeconomic, racial/ethnic, and geographic populations if the health insurance coverage for diagnosis and treatment of lung cancer was consistent across all insurers?"
- ❖ What is the relative contribution of lifestyle, co-morbidities, and care utilization to the relationship between insurance type or benefits and lung cancer outcomes?
- ❖ Does the type of health insurance coverage impact the receipt of guideline therapy/treatment type for patients with lung cancer? If so, does this relationship vary by stage of disease, age, socioeconomic status, or race/ethnicity?
- ❖ What are the differences in patient/provider interactions and treatment decisions in lung cancer patients by insurance type? How is this related to survival differences? Are there differences by urban and rural residence, socioeconomic status, and race/ethnicity?

6. **Would paid FMLA (Family Medical Leave Act) legislation for caregivers benefit lung cancer outcomes and how?**

- ❖ Would paid family medical leave legislation for caregivers improve outcomes for lung cancer patients? Which legislative models are most effective and have the largest impact? Which are most cost effective?
- ❖ How does the impact of family medical leave legislation on lung cancer outcomes vary by caregiver/patient cultural background, immigrant status, employment status, and workplace environment?
- ❖ What is the comparative effectiveness of caregiver support interventions to reduce caregiver burden across the lung cancer care continuum? How do they impact patient outcomes? Does effectiveness vary by caregiver/patient cultural background or socioeconomic status?

7. **If the Primary Care Physician recommended regular screening, the patient met qualifying guidelines, and the cost was covered, would they have the screening? What are the reasons why not? Would it change the lung cancer outcomes?**

- ❖ If Primary Care Physicians recommend lung cancer screenings to eligible patients, and there was no cost, would patients get the screening? If not, what are the contributing factors to their decision? How do these vary by race/ethnicity, socioeconomic status and health literacy?
- ❖ What interventions are effective at increasing compliance with physician lung cancer screening recommendations?
- ❖ What patient care models are effective at providing timely lung cancer screening referrals to all at risk patients? What impact will this have on clinic practice?
- ❖ What are the most effective communication strategies that result in patient compliance with lung cancer screening referrals?

Quality of Care

8. **If we could affect perceptions of care at local hospitals & providers, would it change outcomes? (Quality of care)**

- ❖ What impact does negative perceptions of local healthcare quality have on lung cancer outcomes? How does it have this impact, i.e. does it result in care seeking delay, delay of diagnosis or treatment?
- ❖ Does an improvement in community perceptions of local healthcare result in improved lung cancer outcomes? How? How does this differ by race/ethnicity, socioeconomic status, and geographic location (rural/urban)?
- ❖ What aspects of healthcare provision are most important to perceptions of care? What are effective strategies to improve patient satisfaction and healthcare provider reputation that would impact lung cancer outcomes?

9. **Would more assistance navigating the healthcare system improve outcomes? (Quality of care)**

- ❖ Would more assistance navigating the healthcare system improve lung cancer outcomes including time to treatment, symptom management, quality of life, and survival?
- ❖ Which patient navigation models are most effective at improving lung cancer survival, quality of life, and symptom management outcomes? Does this vary by geographic location (rural, suburban, urban)? Does this vary by socioeconomic status, literacy, race/ethnicity?
- ❖ How important is time from detection of lung abnormalities to cancer treatment as a predictor of lung cancer survival rates?
- ❖ Which navigation models provide the greatest cost effectiveness to lung cancer patients and healthcare facilities?

Social Determinants of Health

10. **Does living in a poverty stricken area vs an area of higher standards affect lung cancer? (Social determinants of health)**

- What individual and community characteristics of low SES neighborhoods negatively impact lung cancer stage of diagnosis, timely treatment and survival? What impact does food insecurity, housing instability, and healthcare access have?
- What barriers prevent people living in low SES areas from seeking timely care, particularly African Americans and other minority populations?
- What strategies can be implemented to improve lung cancer outcomes in patients of low socioeconomic status? Can pre and post treatment assessment of social and behavioral risk factors of patients with low socioeconomic status improve lung cancer outcomes?
- Would increased prevention efforts impact lung cancer outcomes in the homeless population?

11. **If we could improve the general health of the population would it affect lung cancer outcomes? (Social determinants of care)**

- Would an improvement in overall population health, including a reduction in obesity, chronic disease, and smoking prevalence affect lung cancer incidence and outcomes? How does that vary by socio-economic status, race/ethnicity, place of residence, and gender?
- What specific dietary patterns and foods are associated with a reduction in lung cancer incidence and improvement in lung cancer treatment outcomes? Will dietary interventions decrease lung cancer incidence and improve treatment outcomes?
- What levels of physical activity are required for a reduction in lung cancer incidence, and how does this vary by lifestyle (i.e. smoking status) and gender status. Will physical activity interventions decrease lung cancer incidence and improve treatment outcomes?
- What impact does the presence of co-morbid conditions in lung cancer patients, including diabetes, heart disease, and obesity, have on treatment received and lung cancer outcomes? Would effective treatment of co-morbid conditions improve lung cancer treatment options and outcomes?

Support Systems/Coping Mechanisms

12. **What are the factors of patient's faith (for example, knowing what happens when you die, feeling of peace or seeing family members again) and knowing family and community are praying for them; and how does this affect lung cancer outcomes? Does it reduce stress and does it change their outlook? (Support Systems/Coping Mechanisms)**

- How does religious faith affect lung cancer patients' decision making about their treatment options and health care?
- How do patient lung cancer outcomes differ between people with and without faith?
- What health and quality of life outcomes are impacted by religious faith among patients with lung cancer, including stress and survivorship?
